# Obituary of Dr. Julio Toporovski

**DOI:** 10.1590/2175-8239-JBN-2024-IM003en

**Published:** 2024-08-19

**Authors:** Maria Helena Vaisbich, Olberes Vitor Braga de Andrade, Maria Goretti Moreira Guimarães Penido

**Affiliations:** 1 Universidade de São Paulo, Faculdade de Medicina, São Paulo, SP, Brazil.; 2 Santa Casa de São Paulo, São Paulo, SP, Brazil.; 3 Santa Casa de Belo Horizonte, Unidade de Nefrologia Pediátrica, Belo Horizonte, MG, Brazil.

On March 17^th^, 2024, the pediatric and nephrology communities lost one of their most eminent members, Professor Dr. Julio Toporovski, who passed away at the age of 93. Acknowledged as one of Brazil’s leading pediatricians, he dedicated more than six decades of his life to caring for thousands of children and adolescents, leaving an invaluable legacy.

Fondly known as “Dr. Julio”, he was a respected and admired individual, and was the pediatrician of choice for many Brazilian families, especially in São Paulo. His charismatic leadership and exceptional knowledge in the field of pediatric nephrology distinguished him as a pioneer in his specialty, both nationally and internationally.

He was born in São Paulo to a Jewish family. He grew up and attended elementary and middle school in the Bom Retiro neighborhood until 1948. In his youth, already in the company of Dona Dora, his future wife, he participated in a Jewish center for cultural activities, closely aligned with the movements of the left-wing political parties at the time. He completed his medical degree at the Escola Paulista de Medicina from 1949 to 1955, where he became involved in the student movements of the time.

In 1956, he joined the Pediatric Clinic of Santa Casa de Misericórdia in São Paulo, where he undertook roles in care, organization and teaching at the Children’s Emergency Department, Pediatric Clinic, Academic Center, Neonatal Nursery, and the third-floor hospital ward. In January 1966, he organized and started coordinating the Nephrology Unit within the Pediatrics Department, as well as the first outpatient activities in the field.

In addition to his pioneering work in pediatric nephrology, Dr. Julio was one of the first to perform percutaneous kidney biopsies in children back in 1966. For a long time, before the era of interventional radiology, the Pediatric Nephrology Unit performed three to five kidney biopsies on a weekly basis.

Throughout his academic career, he held several roles in undergraduate and postgraduate teaching, participated in numerous examining committees, organized and chaired congresses, among other scientific activities^1,2,3,4^.

He was involved in the training of countless residents and interns, general pediatricians, pediatric nephrologists, and clinical nephrologists with a special interest in learning the specialty. He interacted with nephrologists from all over Brazil and abroad, especially from Latin America, where they received their training at the Pediatric Nephrology Department of Santa Casa de São Paulo.

In 1974 he defended his Habilitation, and in 1977 he became the first Brazilian nephrologist to participate in the International Study of Kidney Disease in Children, helping to standardize the treatment of nephrotic syndrome and other glomerulopathies.

He actively took part in several medical societies and associations. Noteworthy was his involvement in the São Paulo Pediatric Society, the Brazilian Society of Pediatrics, the Brazilian Society of Nephrology, the Latin American Association of Pediatric Nephrology (ALANEPE) and the International Pediatric Nephrology Association (IPNA).

He was president of the São Paulo Pediatric Society from 1987 to 1989, and during his term, he conceived the *“Programa Mãe Participante”* (Participating Mother Program), a humanitarian legacy for Brazilian society. This program made it compulsory for mothers or a family member to be present during the 24-hour stay of hospitalized children.

**Figure 1:**
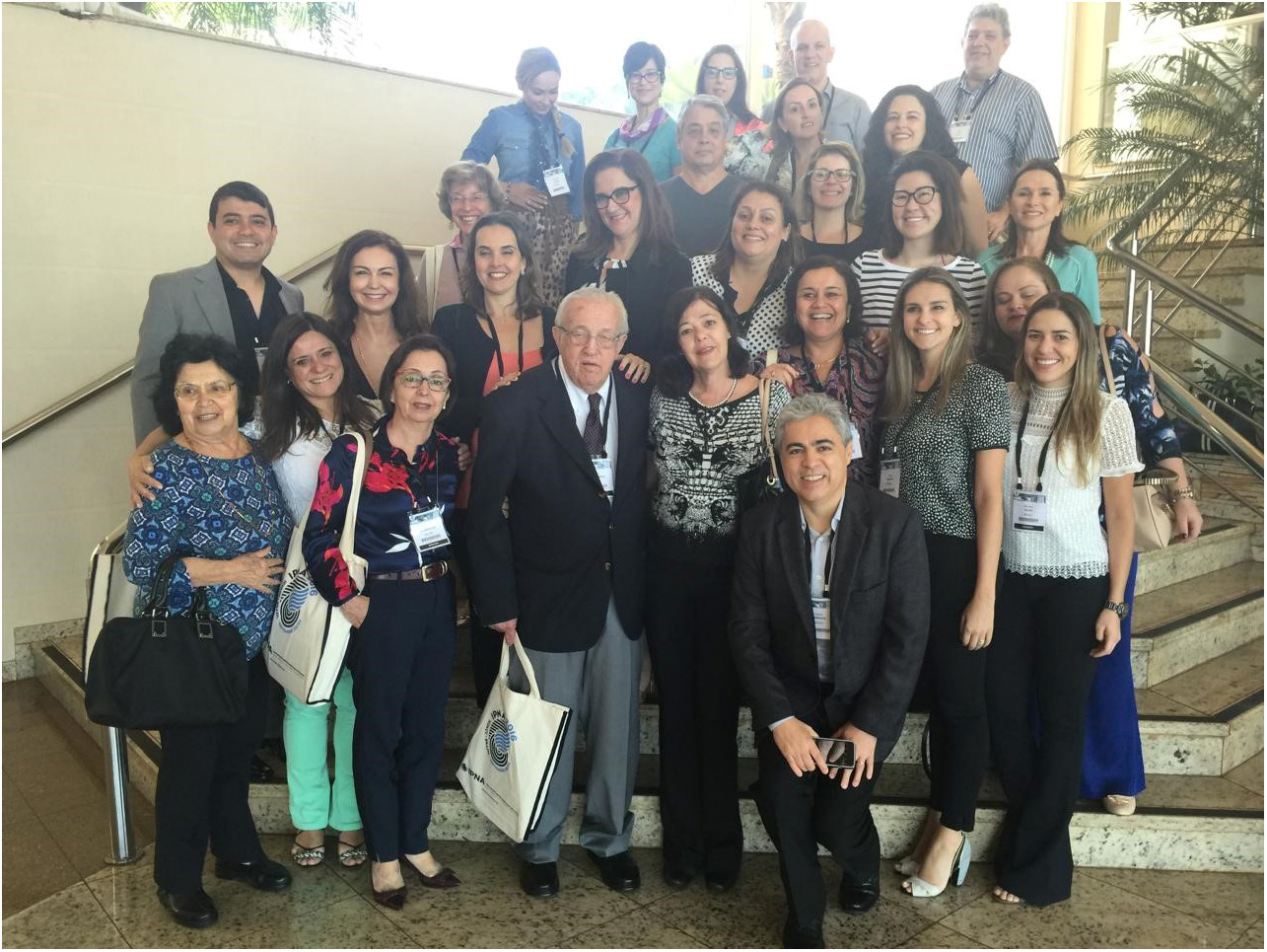
Dr. Julio Toporovski surrounded by several pediatric nephrologists during the 17th Congress of the International Association of Pediatric Nephrology, held in Foz do Iguaçu in 2016. Dona Dora, his wife, is in the lower left corner of the picture.

He received numerous awards during his lifetime, deservedly so, including honors from graduates and honorable mentions from national and international societies, such as the Lifelong Achievement Award in 2016 from the International Pediatric Nephrology Association. In 2018, Professor Julio was deservingly awarded the academic title of Emeritus Professor by the Faculty of Medical Sciences at Santa Casa de São Paulo (Figure 1).

With his national and international scientific contributions, Professor Julio was one of those responsible for placing the Brazilian pediatric nephrology in the global context of the main services^5–10^. Another great virtue was the development of an interface among university centers, fostering integration between professionals in pediatric nephrology and adult clinical nephrology. This relationship has allowed for greater development and training in our specialty.

Dr. Julio was austere, demanding punctuality and proper medical conduct from those around him, and also concerned with the teaching and learning of the younger ones. Despite this apparent solidity, he also had his human moments of concern for the less fortunate, an omnipresent profile of patients and their families in the public healthcare system.

And often, this Corinthians fan was witty and playful. Some of his sayings are well-known among those who worked with him: “In the end, it all boils down to this”; “In this biopsy, all cats are grey”, and “Whoever has one kidney is safe”. “Who’s paying for coffee today?” or “There’s nothing left here” were the hallmarks of relaxation moments during hospital breaks or social gatherings.

In his personal life, Dr. Julio was a man of simple preferences. He enjoyed music, movies and reading. He was always very close to and proud of his family, of spending time with his wife, his children, Mauro (a physician and professor at Santa Casa de São Paulo), Jairo, and Rochele, as well as his six grandchildren and six great-grandchildren.

He was always keeping himself up to date, and his generosity extended to offering articles to younger professionals, similar to what colleagues in the health area currently do in groups and on social media, disseminating scientific knowledge.

His dedication to case studies, constant scientific updating, and comprehensive care for patients and their families were and will always be an inspiration to those who had the honor of having Dr. Julio as a mentor and witnessing his work in Medicine.

When special people enter our lives for good, they teach us lessons that last a lifetime. They teach us about fraternity, compassion, wisdom, understanding, forgiveness, and how to grow, learn, and teach. Sometimes these special people pass away and leave behind a lesson that should always be passed on and acted upon. For these and many other reasons, Dr. Julio will continue to be part of our lives. His legacy will endure through generations, inspiring those who follow in the footsteps of medicine, pediatrics, and pediatric nephrology.
