# Supplementary material for: Obituary of Dr. Julio Toporovski
Source: J Bras Nefrol. 2024 Aug 19;46(3):e2024IM003. doi: 10.1590/2175-8239-JBN-2024-IM003en (PMC11334940; doi:10.1590/2175-8239-JBN-2024-IM003en)
Supplement: Translation

# Translation: Obituário do Dr. Julio Toporovski

**DOI:** 10.1590/2175-8239-JBN-2024-IM003pt

**Authors:** Maria Helena Vaisbich, Olberes Vitor Braga de Andrade, Maria Goretti Moreira Guimarães Penido

**Affiliations:** 1 Universidade de São Paulo, Faculdade de Medicina, São Paulo, SP, Brasil.; 2 Santa Casa de São Paulo, São Paulo, SP, Brasil.; 3 Santa Casa de Belo Horizonte, Unidade de Nefrologia Pediátrica, Belo Horizonte, MG, Brasil.

Em 17 de março de 2024, a comunidade pediátrica e nefrológica perdeu um de seus mais eminentes membros, o Professor Dr. Julio Toporovski, que faleceu aos 93 anos de idade. Reconhecido como um dos principais pediatras do Brasil, dedicou mais de seis décadas de sua vida ao cuidado de milhares de crianças e adolescentes, deixando um legado inestimável.

Afetuosamente conhecido como “Dr. Julio”, ele era uma figura respeitada e admirada, e foi o pediatra de confiança para muitas famílias brasileiras, especialmente em São Paulo. Sua liderança carismática e seu conhecimento excepcional em nefrologia pediátrica o destacaram como pioneiro em sua especialidade, tanto nacional quanto internacionalmente.

De família judaica, nasceu em São Paulo. Cresceu e estudou o primário e o ginásio no bairro do Bom Retiro até 1948. Na juventude, já em companhia da Dona Dora, sua futura esposa, participava de um centro judaico de atividades culturais, muito em consonância com os movimentos dos partidos de esquerda naquele tempo. Sua graduação médica foi na Escola Paulista de Medicina, entre 1949 e 1955, onde se envolveu com movimentos estudantis da época.

Em 1956, vinculou-se à Clínica Pediátrica da Santa Casa de Misericórdia de São Paulo e exerceu funções assistenciais, organizacionais e didáticas no Pronto Socorro Infantil, Clínica Pediátrica, Centro Acadêmico, Berçário de Prematuros e na Enfermaria do terceiro andar. Em janeiro de 1966, organizou e iniciou a coordenação da Unidade de Nefrologia do Departamento de Pediatria e as primeiras atividades ambulatoriais da especialidade.

Além da vanguarda na área da nefrologia pediátrica, Dr. Julio foi um dos pioneiros na realização de biópsia renal percutânea em crianças nos idos de 1966. Por um longo período, antes da era da radiologia intervencionista, a Unidade de Nefrologia Pediátrica chegava a realizar, semanalmente, de três a cinco biópsias renais.

Ao longo de sua carreira acadêmica, exerceu várias funções de ensino de graduação, pós-graduação, participação em inúmeras bancas examinadoras, organização e presidência de congressos, entre outras atividades científicas^1–4^.

Participou na formação de inúmeros residentes e estagiários, pediatras gerais, nefrologistas pediátricos e nefrologistas clínicos com interesse no aprendizado da especialidade. Interagiu com nefrologistas oriundos de todo o Brasil e do exterior, principalmente da América Latina, onde realizavam seu treinamento na Nefrologia Pediátrica da Santa Casa de São Paulo.

Em 1974 defendeu sua Livre Docência e em 1977 foi o primeiro nefrologista brasileiro com participação no *International Study of Kidney Disease in Children,* auxiliando na padronização do tratamento da síndrome nefrótica e de outras glomerulopatias.

Participou ativamente de várias sociedades e associações médicas. Destaca-se sua atuação na Sociedade Paulista de Pediatria, Sociedade Brasileira de Pediatria, Sociedade Brasileira de Nefrologia, Associação Latino-Americana de Nefrologia Pediátrica (ALANEPE) e Associação Internacional de Nefrologia Pediátrica (IPNA).

Foi presidente da Sociedade de Pediatria de São Paulo entre 1987 e 1989 e, durante sua gestão, foi o idealizador do “Programa Mãe Participante”, um legado humanitário para a sociedade brasileira, tornando obrigatória a presença das mães ou de um acompanhante familiar durante as 24 horas de internação das crianças hospitalizadas.

Recebeu, merecidamente, várias premiações em vida, homenagens de formandos e menções honrosas oferecidas por sociedades nacionais e internacionais, como o *Lifelong Achievement Award* em 2016, pela *International Pediatric Nephrology Association*. Em 2018, o professor Julio merecidamente recebeu a titulação acadêmica de Professor Emérito pela Faculdade de Ciências Médicas da Santa Casa de São Paulo (Figura 1).

**Figura 1:** Dr. Julio Toporovski rodeado por vários nefrologistas pediátricos durante o 17o Congresso da Associação Internacional de Nefrologia Pediátrica, realizado em Foz do Iguaçu em 2016. Dona Dora, sua esposa, se encontra no canto inferior esquerdo da imagem.

Com suas contribuições científicas nacionais e internacionais, o professor Julio foi um dos responsáveis por situar a nefrologia pediátrica nacional no contexto mundial dos principais serviços^5-10^. Outra grande virtude foi a elaboração de uma interface entre os centros universitários, estimulando a integração entre os profissionais da nefrologia pediátrica e da nefrologia clínica de adultos. Esse vínculo permitiu um maior desenvolvimento e capacitação da nossa especialidade.

Dr. Julio era austero, exigente com a pontualidade e com a boa conduta médica dos que o cercavam, e também preocupado com o ensino e aprendizado dos mais jovens. Apesar dessa solidez aparente, ele tinha seu momento humano de preocupação também com os mais carentes, perfil onipresente dos pacientes e familiares no serviço público.

E muitas vezes esse corintiano era espirituoso e brincalhão. Alguns jargões dele são conhecidos por quem convivia na área profissional: “No frigir dos ovos”; “Nesta biópsia, todos os gatos são pardos” e “Quem tem um rim, tá salvo”. “Quem paga o café hoje?” ou “Aqui não dá mais nada” eram marcas de momentos de relaxamento nos intervalos do hospital ou em reuniões sociais.

Na vida pessoal, Dr. Julio era um homem de gostos simples. Apreciava música, cinema e leitura. Sempre esteve muito próximo e demonstrava orgulho de seus familiares, do convívio com a esposa, os filhos, Mauro (médico e professor na Santa Casa de São Paulo), Jairo e Rochele, e os seis netos e seis bisnetos.

Sempre estava se atualizando, e sua generosidade se estendia no oferecimento de artigos aos mais jovens, similar ao que os amigos da área de saúde fazem hoje em grupos e mídias sociais, disseminando o conhecimento científico.

Sua dedicação aos estudos dos casos, a constante atualização científica, a atenção integral aos pacientes e suas famílias foram e sempre serão uma inspiração para aqueles que tiveram a honra de terem tido Dr. Julio como mestre e acompanhar sua atividade na Medicina.

Quando pessoas especiais entram definitivamente em nossas vidas, elas ensinam lições para uma vida inteira. Ensinam o que é fraternidade, compaixão, sabedoria, compreensão, perdão e como crescer, aprender e ensinar. Às vezes, essas pessoas especiais morrem e deixam uma lição que deverá sempre ser repassada e executada. Por essas e muitas outras razões, Dr. Julio continuará presente em nossas vidas. Seu legado perdurará através das gerações, inspirando aqueles que seguem os passos da medicina, da pediatria e da nefrologia pediátrica.
